# Supplementary figures and images for: Nostolepis scale remains (stem Chondrichthyes) from the Lower Devonian of Qujing, Yunnan, China
Source: PeerJ. 2021 May 7;9:e11093. doi: 10.7717/peerj.11093 (PMC8109008; doi:10.7717/peerj.11093)

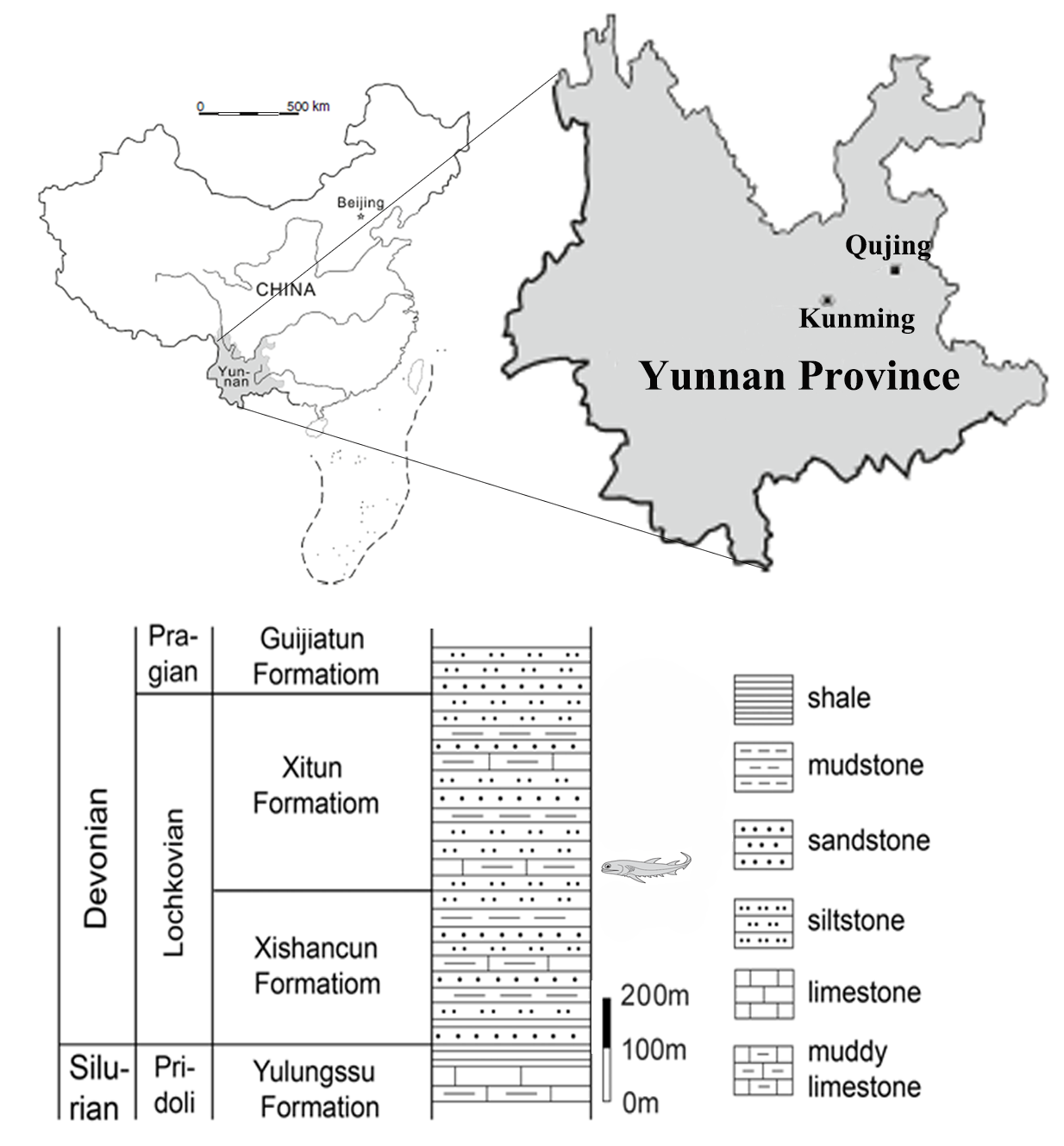

Supplement: Supplemental Information 1 [file peerj-09-11093-s001.png]
